# The total flavonoids from *Selaginella tamariscina* (beauv.) Spring improve glucose and lipid metabolism in db/db mice

**DOI:** 10.22038/ijbms.2020.40532.9594

**Published:** 2020-10

**Authors:** Xiaolan Wang, Aozi Feng, Peipei Yuan, Yang Fu, Zhiyao Bai, Ning Zhou, Xiaoke Zheng

**Affiliations:** 1 Henan University of Chinese Medicine, Zhengzhou, China; 2 The Engineering and Technology Center for Chinese Medicine Development of Henan Province, Zhengzhou, China; 3 First Affiliated Hospital, Jinan University, Guangzhou, China

**Keywords:** Biomarker, Flavonoids, Glucose metabolism- disorders, Glucose transporter type 4, Lipid metabolism disorders, 3-Phosphoinositide-dependent protein kinases, PPAR gamma, Selaginellaceae

## Abstract

**Objective(s):**

This study aimed to investigate the glucose and lipid metabolism improving effect of the total flavonoids from *Selaginella tamariscina* (Beauv.) Spring (TFST) on db/db mice, and to study its mechanism of action.

**Materials and Methods:**

The db/db mice were divided into 5 groups: the normal group (NC), the diabetic group (DM), the gliclazide group (GZ), the DM+TFST (110 mg/kg), and the DM+TFST (220 mg/kg). The body weight, blood glucose, INS, GC, TC, TG, LDL, and HDL were detected. HE staining was used to observe the liver and pancreas. Urine was tested by UPLC-QTOF-MS to study the metabolic differences of each group, coupled with SIMCA-P13.0 for PCA and OPLS-DA analysis, to identify potential biomarkers, find the metabolic pathway. Western blot was used to examine liver tissue of mice for studying effect of TFST on the PPAR-γ/PI3K/GLU4 pathway.

**Results:**

TFST can reduce the weight and levels of TC, TG, and LDL-C, increase the level of GC in blood, and reduce the fat accumulation and inflammation in the liver, and repair the islet cell. 13 biomarkers were identified, they are mainly involved in amino acid metabolism, and purine and pyrimidine metabolism. The results of Western blot show TFST can improve the utilization rate of GLU4 by regulating PPAR-γ and PI3K expression in the liver of db/db mice.

**Conclusion:**

TFST can improve glucose and lipid metabolism of DM, which relates to regulation of the PPAR-γ/PI3K/GLU4 signaling pathway, and affect the amino acid metabolism, purine, and pyrimidine metabolism.

## Introduction

The pathogenesis of type 2 diabetes (T2DM) is complicated by many factors, characterized by chronic hyperglycemia and hypoinsulinemia. T2DM affects multiple organs and tissues throughout the body and is a systemic metabolic disease (1). In the T2DM patients, the disorder of glucose metabolism accelerates disturbances in lipid, amino acid, and energy metabolism (2, 3). Therefore, detection of endogenous metabolic variations in the entire body as T2DM developing and progressing becomes highly significant. Metabonomics can provide a lot of information on disease early diagnosis and processes, drug toxicity and gene function, and has shown great potential in diabetes research (4, 5).

*Selaginella tamariscina *(Beauv.) Spring has long been used to treat blood stagnation in traditional Chinese medicine and many previous studies have focused on its effect on the cardiovascular system and metabolic syndrome(6,7). Total flavonoids from *Selaginella tamariscina *(Beauv.) Spring (TFST) are hypoglycemic effective fractions of this medicinal plant (8, 9). The main component of TFST is amentoflavone (AF) that has various functions, such as regulation of glycolipid metabolism(10-12), improvement of insulin resistance and liver steatosis (13), anti-oxidative (14) and anti-inflammatory effect (15). Our preliminary results show that TFST has an insulin-sensitizing activity which can increase the expression of PPAR-γ in the muscles of type 2 diabetic rats (9). However, little is known about the effects of TFST on insulin signal transduction in diabetic mice through PPAR-γ/ PI3K/GLU4 signal transduction pathway, and metabolic markers and metabolic pathways.

In this study, we used db/db mice as a spontaneous diabetic model to investigate the improving glucose and lipid metabolism effects of TFST by focusing on the morphology of the liver and pancreas. The urine was tested by UPLC-QTOF-MS to study the metabolic differences of each group, and research the expression of key proteins involved in PPAR-γ/PI3K/GLU4 pathway in the liver. The finding will help understand the mechanism for the anti-hyperglycemic activity of TFST in spontaneously diabetic animals. 

## Materials and Methods

***Materials***

TFST with 58% flavonoids was purchased from Kanglu Biotechnology Co., Ltd, Hunan, China. Gliclazide tablets were purchased from Jingfeng pharmaceutical group co., Ltd, Beijing, China. The glucose detection kit was purchased from Zhongsheng Beikong Biotechnology Co., Ltd. The mouse insulin (INS), mouse glucagon (GC) enzyme-linked immunosorbent assay kit, mouse glucagon (GC) enzyme-linked immunosorbent assay kit, triglyceride TG, TC, LDL-C, and HDL-C test kit were purchased from Nanjing Jiancheng Bioengineering Institute, Nanjing, China. Antibodies PPAR-γ and β-actin were purchased from Proteintech^TM^ Biotechnology Co., Ltd, Wuhan, China. PI3K and GLU4 were purchased from Bioss Biotechnology Co., Ltd, Beijing, China. 

***Animals***

Six-week-old male db/db mice and db/m mice were purchased from the Changzhou Cavans Experimental Animal Co., Ltd. All mice were kept in an IVC animal experiment system (temperature: 23±2 ^°^C, relative humidity of the air: 40%-70%, and a 12 hr light/12 hr dark cycle), and were fed standard rodent chow and water. Twenty-four mice were randomly assigned into four groups of 6 mice per group. Mice in the diabetic control group (DM) were orally administered 2 ml of water. Mice in the gliclazide group (GZ) were orally administered gliclazide (24 mg/kg) as positive control. Mice in treatment groups were orally administered high-dose total flavonoids (TFSTH, 220 mg/kg) and the low-dose total flavonoids group (TFSTL, 110 mg/kg) for 3 weeks. Meanwhile, 6 db/m mice were treated with the same volume of water as normal control (NC). All animal procedures and protocols were approved by the Institutional Animal Care and Use Committee of Henan University of Traditional Chinese Medicine.

***Determination of body weight***

Body weights of mice in each group were recorded at 0, 1, 2, and 3 weeks following initiation of treatment, respectively.

***Biochemical assays***

The blood was taken from the tail tip of mice in each group 1 week, 2 weeks, and 3 weeks after administration, respectively, and blood glucose levels were measured with a glucometer (Omron Electronics, China). The levels of INS, GC, TC, TG, LDL-C, and HDL-C were measured using commercial assay kits according to the manufacturer’s instructions.

***Pathological observation of liver and pancreas***

After 3 weeks of administration, mice were weighed, and blood samples were taken from the veins after anesthesia. Mice were then sacrificed, the liver and pancreas were dissected and fixed with 10% neutral formaldehyde for 48 hr. Paraffin sections were prepared and stained with hematoxylin and eosin (HE).

***Metabolomics analysis***

Urine sample preparation and analysis: Urine samples were thawed in ice-water and then centrifuged at 4 ^°^C for 10 min (3500 r/min). Each 500 µl supernatant was mixed with 1500 µl cold acetonitrile. The mixture was vortexed for 3 min and centrifuged for 10 min (12000 r/min), then 1 ml supernatant in sample vials for UPLC-Q/TOF-MS analysis.

UPLC-QTOF-MS analysis of urine: Urine samples were separated using an Acclaim RSLC 120 C18 column (2.1×100 mm, 2.2 μm) on a Dionex UltiMate 3000 UPLC System (Thermo Scientific, USA) and then screened using ESI-MS. The mobile phase was composed of 0.1% formic acid in water (solvent A) and 0.1% formic acid in acetonitrile (solvent B) with gradient elution (0~1 min, 2~10%B; 1~9 min, 10~20%B; 9~16 min, 20~30%B; 16~20 min, 30~98%B). The flow rate was 0.3 ml/min, and the column temperature was maintained at 40 ^°^C.

Mass detection was performed on a MaXis HD quadrupole time-of-flight mass spectrometer (QTOF-MS) (Bruker, Bremen, Germany) using an electrospray ionization (ESI) source. The scanning range was from 50 to 1000 m/z at a spectra rate of 1.00 Hz. The capillary voltage was maintained at 3500 V. The pressure of the nebulizer (nitrogen) was set at 2.0 bar. The temperature and flow rate of the dry gas temperature was set at 230 ^°^C and 8 l/min, respectively. The flow velocity was set at 48 µl/hr.

***Western blot***

Total proteins were extracted from the liver using a protein extraction kit (BeijingCW Biotech Co. Ltd., Beijing, China). Protein concentrations were determined with the Bradford protein assay kit (Wuhan Boster Biological Technology Ltd, Wuhan, China). Proteins were separated by SDS-PAGE and electrophoretically transferred to the PVDF membrane. The membrane was blocked with 5% non-fat milk in Tris-buffered saline supplemented with 0.5% Tween 20 (TBST) for 2 hr at room temperature and then incubated with specific primary antibodies, including anti-PPARγ (1:1000), anti-PI3K (1:1000), anti-GLU4 (1:1000), and β-actin (1:3000), overnight at 4 ^°^C. After washing with TBST three times, the membrane was incubated with a secondary antibody (1:3000) for 1 hr at room temperature. β–actin was used as the reference protein. The intensity of the protein bands was quantified using Gene Tools.

***Statistical analysis***

Raw data were corrected by subtracting background noise and aligning peaks and then normalized using ProfileAnalysis 2.1 (Bruker, Germany). Principal component analysis (PCA) was performed using SIMCA-P 13.0 (Umetrics AB, Muea, Sweden) and potential biomarkers were identified using orthogonal partial least squares discriminant analysis (OPLS-DA). The biomarkers were filtered by the results of variable importance for the projection (VIP) values (VIP>4, and *t*-test *P*<0.05). Statistical analysis was performed using one-way ANOVA (SPSS 18.0, IBM, New York, NY, USA). *P*<0.05 was accepted as significant.

## Results

***Effects of TFST on body weight of db/db mice***

**Figure 1:**
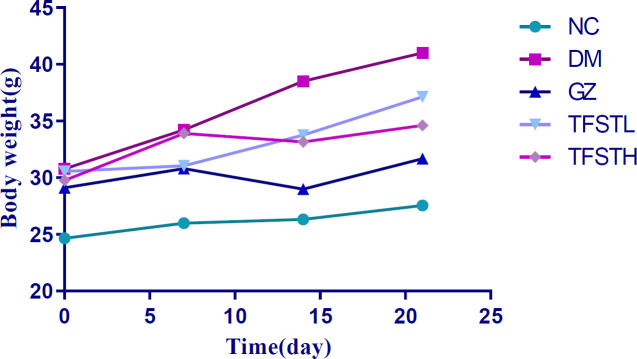
Body weight from animals of different groups

FST treatment did affect the body weights of db/db mice, as shown in Figure 1. The NC group grew normally and did not gain much body weight during 3 weeks. On the other hand, though db/db mice in the DM group had similar body weights as db/m mice in the NC group (*P*>0.05) at the beginning of the experiment (0 weeks), they gained excessive body weights during 3 weeks that were significantly higher than those of db/m mice at the end of each week (*P*<0.01). After 2 and 3 weeks of gliclazide treatment, the body weights of db/db mice in the GZ group did not increase and were significantly lower than those of the DM group (*P*<0.01). Interestingly, TFSTL could slow the weight gain of db/db mice, which was significantly lower than that of the DM group (*P*<0.05). After 2 and 3 weeks of administration, TFSTH and TFSTL could alleviate the excessive weight gain of db/db mice, and TFSTL-treated db/db mice had better weight control (Figure 1).

***Biochemical analysis***

**Figure 2:**
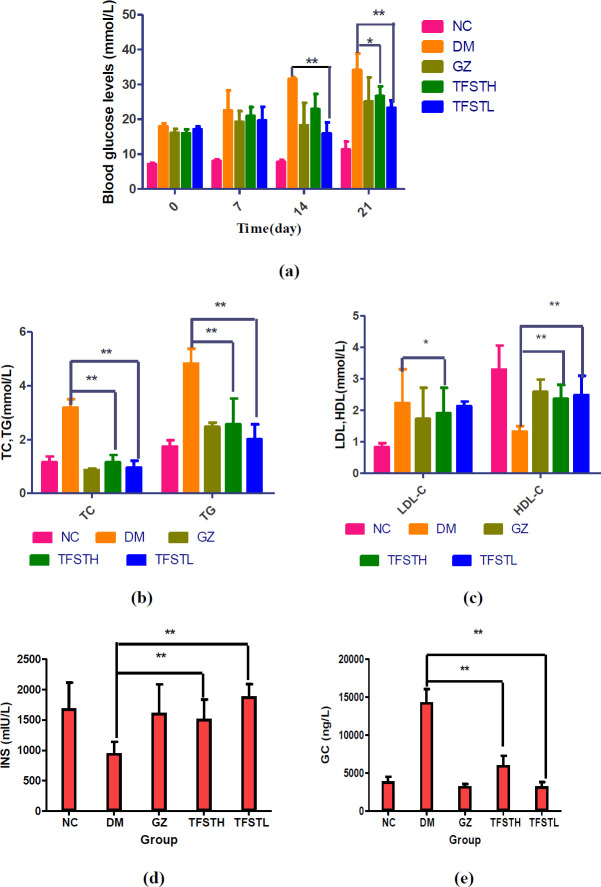
Serum levels of random blood glucose (a), TC and TG (b), LDL-C and HDL-C (c), INS (d), GC (e), * *P<*0.05; ** *P<*0.01 vs DM group

As shown in Figure 2a, before administration, the blood glucose levels of the NC group were significantly lower than the DM group (*P*<0.05). The blood glucose of the DM group was increased gradually and significantly higher than the NC group. After 2 and 3 weeks, the blood glucose levels of TFSTL were significantly lower than DM, and low dose of TFST showed a better effect on reducing the fasting blood glucose level, reduced the levels of TC, TG, LDL-C, GC levels, at the same time increased the levels of HDLC and INS in the blood of db/db mice (*P*<0.05 or *P*<0.01), as shown in Figure 2b, d.

***Pathological observation of liver and pancreas in db/db mice after TFST treatment***

**Figure 3:**
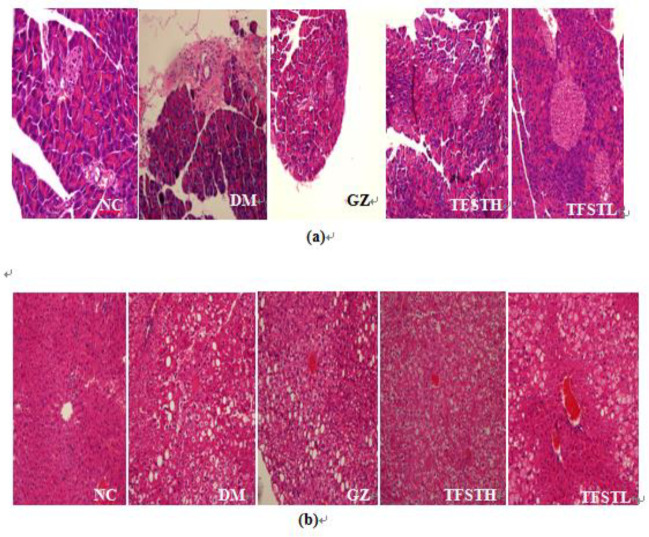
Pathological observation of pancreata and livers in db/db mice after TFST treatment. The tissues were stained with hematoxylin and eosin (H&E) and observed using a light microscope (magnification: 200×)

The primary target organs for insulin were the pancreas and liver, and the pathomorphological features of these tissues after TFST treatment were observed, as shown in Figure 3. 

Pancreas: Pancreatic cells in the normal group are evenly distributed, the islets are structurally intact, the edges are clear. According to the comparison of the group, the pancreatic tissue of the model group showed hyperemia, acinar dilatation, disordered islet cell arrangement, decreased number, and even cell vacuolization. The TFST group had different degrees of improvement of islet cell arrangement, tissue hyperemia, and cell vacuoles. As shown in Figure 3a.

Liver: The liver tissue of the NC group was intact and clear, the hepatic cells were arranged regularly and had rounded large nuclei, the cytoplasm was uniform, and the hepatic sinusoidal structure was not changed. In the DM group, the hepatic lobules of the mice were unclear, and the hepatocytes were steatotic and disordered, and had a large number of accumulated lipid droplets in the liver. After 3 weeks of TFSTL treatment, lipid droplets and vacuoles were significantly reduced in the TFST group. As shown in Figure 3b.

***Metabolomics study***

*PCA analysis*

**Figure 4:**
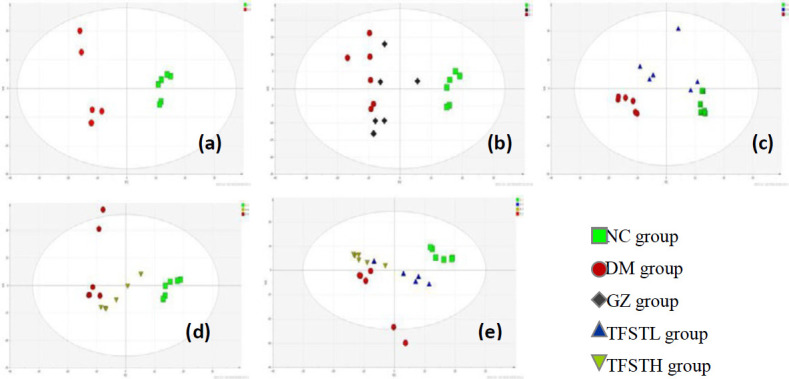
PCA score scatter plots of metabolites obtained from the control model and various treatment groups. (a) NC and DM groups, (b) NC, DM and GZ groups, (c) DM and TFSTL groups, (d) NC, DM and TFSTH groups, and (e) NC, DM, TFSTH, and TFSTL groups

Samples were run in the positive ionization mode monitoring the entire sequence over the stability of the LCMS system, as shown in Figure 4. The samples from the normal group and the model group were divided into two groups, and the separation trend is significant, which indicates the diabetes model is successful, as shown in Figure 4a. The TFSTH and TFSTL metabolic profiling distinctly separate from DM, and assemble to NC, which indicates that the TFST has the effect of improving the metabolic disorder in db/db mice, as shown in Figure 4c, d, and the TFSTL has the better effect as shown in Figure 4e.

*Potential biomarkers*

**Figure 5:**
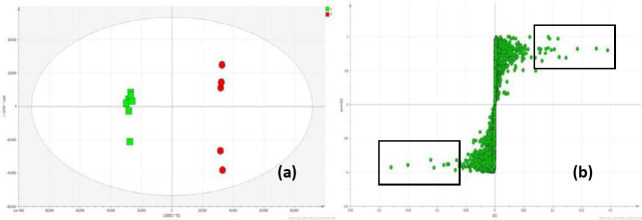
OPLS-DA score scatter plots of metabolites obtained from NC and DM groups (a) and S-plot generated from the OPLS-DA for DM model group (b). The boxes in (b) refer to metabolites with significant changes in urine

The supervised OPLS-DA model was established in order to compare the metabolic differences between NC and DM groups. The OPLS-DA score scatter plot showed a clear separation between the first two components (*R*^2^*X*=0.867,* R*^2^*Y*=0.999, and *Q*^2^=0.97), as shown in Figure 5a. Before being selected as potential biomarkers, the metabolites with significant changes were carefully screened based on VIP values (VIP>4) and *t*-test (*P*<0.05), as shown in Figure 5b.

The structures of metabolites were identified based on accurate mass measurements and MS/MS fragmentation patterns by matching to online metabolite databases such as Metlin (https://metlin.scripps.edu/), Human Metabolome Database (http://www.hmdb.ca/), and MassBank (http://www.massbank.jp/). The structures were further confirmed by comparison of the retention times and MS/MS fragmentation patterns with the authentic standards. Consequently, a total of 13 potential biomarker relate to tyrosine metabolism, arginine and proline metabolism, purine metabolism were identified and are listed in ESI, as shown in Table 1.

***The expression of PI3K, GLU4, and PPARγ in rat liver***

**Figure 6:**
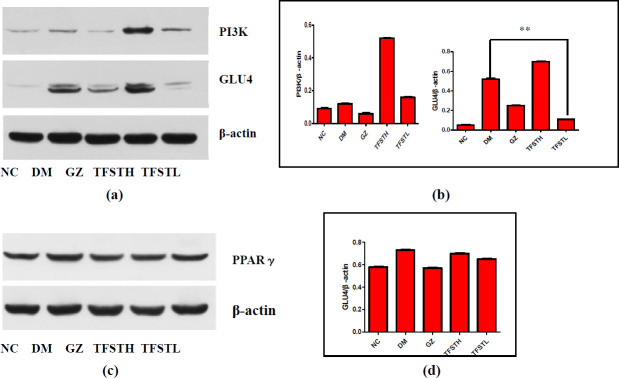
The effect of TFST on PPAR-γ/PI3K/GLU4 pathway

The PPAR-γ and GLU4 protein levels of DM group were significantly increased (*P*<0.05), TFSTL can reduce the increased PPAR-γ expression of the db/db mice, but it is not significant (*P*>0.05), as shown in Figures 6c, d. However, the levels of PI3K proteins of DM were obviously higher than those in NC. TFSTL treatment significantly increased the levels of PI3K proteins in db/db mice, but GZ can reduce the PI3K proteins in the liver as shown in Figures 6a, b, which indicates GZ possibly has carcinogenic risk in treating diabetes (16).

NC: the normal group, DM: the diabetic group, GZ: the gliclazide group, TFSTH : the high dose of TFST group, TFSTL:the low dose of TFST group

NC: the normal group, DM: the diabetic group, GZ: the gliclazide group, TFSTH : the high dose of TFST group, TFSTL:the low dose of TFST group

NC: the normal group, DM: the diabetic group, GZ: the gliclazide group, TFSTH : the high dose of TFST group, TFSTL:the low dose of TFST group

NC: the normal group, DM: the diabetic group, GZ: the gliclazide group, TFSTH : the high dose of TFST group, TFSTL:the low dose of TFST group

* *P<*0.05; ** *P<*0.01 vs DM group

NC: the normal group, DM: the diabetic group, GZ: the gliclazide group, TFSTH : the high dose of TFST group, TFSTL:the low dose of TFST group

**Table 1:** Potential biomarker related to db/db mice

**Number**	**Name**	**Formula**	**Determined** **m/z**	**𝑡**_𝑅_ ** (min)**	**Metabolic Pathway**
1	3-(3,4-Dihydroxyphenyl)pyruvate	C_9_H_8_O_5_	196.0372	10.6	Tyrosine metabolism
2	N4-Acetylaminobutanal	C_6_H_11_NO_2_	129.0790	1.2	Arginine and proline metabolism
3	Phenylacetylglycine	C_10_H_11_NO_3_	193.0739	8.4	Tyrosine metabolism
4	6-Hydroxykynurenate	C_10_H_7_NO_3_	205.0375	6.2	Tryptophan metabolism
5	N-Carbamoylsarcosine	C_4_H_8_N_2_O_3_	132.0535	1	Arginine and proline metabolism
6	(R)(-)-Allantoin	C_4_H_6_N_4_O_3_	158.0440	6	Purine metabolism
7	Creatinine	C_4_H_7_N_3_O	113.0589	1	Arginine and proline metabolism
8	5-Methoxyindoleacetate	C_11_H_11_NO_3_	205.0739	12.8	Tryptophan metabolism
9	L-Methionine	C_5_H_11_NO_2_S	149.0510	1.2	Arginine and proline metabolism
10	Allose	C_6_H_12_O_6_	180.0634	7.2	Fructose and mannose metabolism
11	Uridine	C_9_H_12_N_2_O_6_	244.0695	12.8	Pyrimidine metabolism
12	N-Methylhydantoin	C_4_H_6_N_2_O_2_	114.0429	1.2	Arginine and proline metabolism
13	6-Hydroxykynurenate	C_10_H_7_NO_4_	205.0375	6	Tryptophan metabolism

## Discussion

TFST contains effective hypoglycemic extracts from *Selaginella tamariscina *(Beauv.) Spring and amentoflavone is the main component of TFST (17). TFST also contains other kinds of flavonoids such as hinokiflavone and isocryptomerin. It is reported that 80% ethanol extract of *Selaginella tamariscina* (Beauv.) Spring had the strongest hypoglycemic activity in HepG2 and 3T3-L1 cells and in STZ-induced diabetic rats (8). The mechanism of the hypoglycemic activity of TFST may include inhibition of fat droplet formation in the liver, restoration of normal liver lipid metabolism, and enhancement of insulin sensitivity (11). In addition, *in vitro* cell experiments showed that flavonoids can improve insulin resistance by activating PI3K-Akt insulin signaling (10, 13) However, it is still unknown whether TFST has hypoglycemic activity in spontaneous diabetic animals, as well as the underlying mechanism. Based on our previous studies, spontaneous diabetic db/db mouse was used to study the hypoglycemic activity of TFST and the possible signal transduction pathway. In the study of T2DM metabolomics, the potential markers affect the metabolism, which indicates a potential effect of nutrition metabolism, energy metabolism, and bile acids metabolism by the TCA cycle (18-21). In a previous study, we found that the TFST can significantly change trends of the biomarkers identified, which were related to the action mechanism of TFST. The results indicate it is related to glucose metabolism, amino acid metabolism, and nucleic acid metabolism.

***Tyrosine metabolism***

Tyrosine, a non-essential amino acid, transformed from phenylalanine hydroxylation, catalysis into 4-hydroxy benzene pyruvic acid (HPPA) by tyrosine aminotransferase (TAT), and HPPA oxidization into alkapton. Alkapton is transformed to fumaric acid and acetoacetic acid, participates in the citric acid cycle(22), improves energy metabolism (23), affects signal transduction pathways on PI3K/AKT/GLU4 (24, 25), and improves the insulin resistance (26).

***Arginine and proline metabolism***

Arginine is an amino acid having multiple functions, It is not only the precursor of protein synthesis but also the synthesis of nitric oxide (NO), the precursors of polyamine and pyrimidine (27). Arginine affects the release of hormones and is mediated by Arg/m2 Urea and Arg/NO, the two main metabolic pathways that regulate nitrogen balance, immunity, and metabolism, cardiovascular and other system functions (28). There is a “Cit –NO circulation” between these two kinds of metabolic pathways, regulated by arginine succinic acid synthetase (AS), arginine succinic acid lyase (AL), and nitric oxide synthase (NOS)(29). Through the above two main metabolic pathways to regulate the body’s nitrogen balance, NO concentration, thereby affecting the body’s immune, nervous, cardiovascular, and other system functions (30). Patients with diabetes and macrovascular complications have some changes in arginine metabolism related to endothelial dysfunction (31).

***Purine and pyrimidine metabolism***

Purine and pyrimidine metabolism is closely related to syndrome and disorder, such as diabetic nephropathy, gout, and renal disease (32). In the liver of diabetic rats, biosynthesis and metabolism of purine and pyrimidine correlate with the hepatic enzymic activities, insulin can induce an increase in these activities, affecting the activity of glycolysis and pentose phosphatase (33). In the liver, the transcription factor PPARγ promotes metabolic adaptations of lipogenesis and aerobic glycolysis under the control of Akt2 activity (34).

In the present study, we proved that TEST could significantly reduce the levels of TC, TG, and LDL-C, increase the level of HDL-C to a certain extent, and improve the hepatic fat accumulation and reduce fat droplets in db/db mice. The liver plays an important role in regulating blood sugar levels by maintaining the balance between the storage and release of glucose. It is reported that PPAR-γ agonists can reduce glucose production in the liver of type 2 diabetes (35) Besides, we also found that selaginella extract can increase the expression of PPAR-γ in the muscles of streptozotocin-induced type 2 diabetic rats (8). So far, little is known about the PPAR-γ expression of the rat liver, affected by TEST in the selaginella extract. Interestingly, in our study, the PPAR-γ expression of the db/db mice was up-regulated, which is possibly related to the activation of the heat shock response(HSR)(36). TEST can reduce the expression of ectopic GLU4 and activate the insulin-mediated PI3K/p-Akt signaling pathway (37). Possibly, flavonoids can significantly reduce the GLU4 transporter in adipocyte 3T3 to reduce oil droplets in adipocytes (38), thereby improving insulin sensitivity and glucose homeostasis in type 2 diabetic rats (39). In this study, TEST significantly increased the expression of GLU4 in the liver, suggesting that TEST can improve hyperglycemia by promoting the translocation of GLU4. TEST may stimulate GLUT4 transport by down-regulating PPAR-γ and activating the PI3K/p-Akt signaling pathway to improve insulin resistance and reduce the risk of carcinogenicity. It is worth thinking about the relation of GLUT4 and HSR needs to be further explored and improved.

## Conclusion

TFST can improve glucose and lipid metabolism for T2DM and its mechanism was related to regulation of the PPAR-γ/PI3K/GLU4 signaling pathway, and affecting the amino acid metabolism, purine, and pyrimidine metabolism.
